# Perceptional differences in the factors of local acceptance of waste incineration plant

**DOI:** 10.3389/fpsyg.2022.1067886

**Published:** 2022-12-19

**Authors:** Yangsen Huang, Ziqi Zhang, Yanbo Zhang, Zixing Wang

**Affiliations:** ^1^School of Management, Xuzhou Medical University, Xuzhou, China; ^2^School of Marxism, Xuzhou Medical University, Xuzhou, China; ^3^School of Management Engineering and Business, Hebei University of Engineering, Handan, China; ^4^University of Bristol, Bristol, United Kingdom

**Keywords:** locally unwanted land use, local acceptation, waste incineration plant, Q methodology, China

## Abstract

Existing research has documented that public attitudes towards waste incineration plants are determined by various factors, such as risk perception, economic impacts, and social trust. However, the diversity in perceptions within communities hosting waste incineration plants is understudied. Adds to existing knowledge, the present paper employed the Q methodology to examine the perceptions of residents living in the vicinity of a waste incineration plant in Xuzhou, China. The results revealed four perspectives on residents’ perceptions towards waste incineration plants: I do not trust them and feel besieged by risks; I trust local governments but I am unfairly treated; I attach this place a lot but I am unfairly treated; I possess knowledge of waste incineration and feel besieged by risks. Our data show that risk perception, trust perception, and political efficacy perception are underlying reasons for local acceptance of waste incineration plants. The diversified subjectivities we obtained supplement existing literature that quantitatively documents the influencing factors. These findings demonstrate that it is necessary to explicitly consider the deep-seated values and perspectives among hosting residents’ for the siting of the waste incineration plant.

## Introduction

The acceleration of urbanization and the rapid upsurge of urban dwellers in recent decades have led to a sharp increase in municipal solid waste generation in China. Official statistics show that, in 2018, the solid waste output of the top 200 large and medium-sized cities in China was 21.1473 million tons (Ministry of Ecology and Environment of the People’s Republic of China, 2019), around two-thirds of large and medium-sized cities are experiencing the “urban disease” incurred by “waste siege” (He, 2012). Increasing solid waste can cause severe environmental pollution if proper waste management approaches are not employed promptly (Lee et al., 2017). Potential environmental problems such as air and noise pollution may result from poor waste management and the contamination of soil and water resources (Ziraba et al., 2016). Based on these insights, traditional waste management approaches, such as landfills, are gradually being abandoned due to potential adverse impacts on human health, well-being, and ecosystem integrity (Sang et al., 2006).

Incineration stands out as a practical solution and sustainable coping method for municipal solid waste, winning support among policymakers and researchers. Through incineration, not only can the volume of domestic waste be reduced by 50–80%, but also, the harmful substances in the waste can be eliminated in the process of high-temperature incineration. Also, heat resulting from the incineration process can generate electricity (Klein et al., 2001). Given the potential adverse environmental consequences caused by landfills (Xu and Lin, 2020), and the fact that waste composting is not suitable for all types of domestic waste (Renkow and Rubin, 1998), incineration is becoming a promising waste disposal approach. Moreover, incineration could reduce greenhouse gas emissions and enhance the transition toward a circular economy (Ayodele et al., 2017).

**Figure 1:**
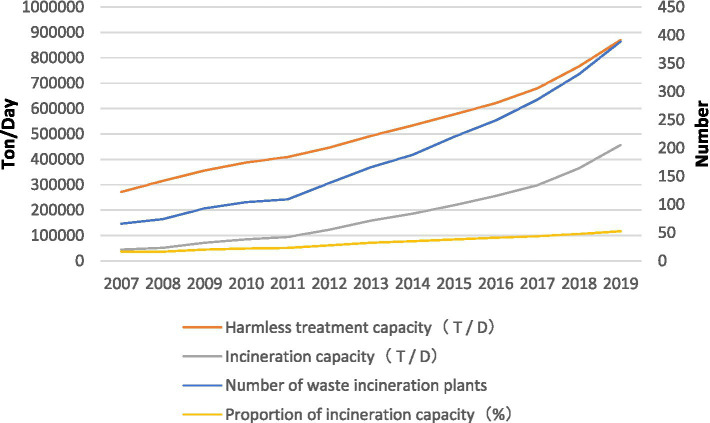
Statistics on waste incineration in China (2007–2019).

Given these advantages, incineration is becoming the foremost choice of municipal solid waste treatment in China (Liu et al., 2006; Cheng and Hu, 2010; Yang et al., 2012). Recent government statistics reveal that incineration capacity for waste disposal has significantly increased since 2010 (Figure 1). By the first quarter of 2019, the number of waste incineration plants in China had exceeded 400, with 178 WIPs under construction and 82 WIPs underway. In 2020, about 600 waste incineration plants were projected to operate (Zhiyan Consulting, 2019).

However, the siting of waste incineration plants in China is challenging local authorities’ governance capacity. Economic orientation has resulted in extensive and expansive urban planning in China. Urban spatial planning lags behind economic development seriously, including unscientific and reasonable use of land resources, unfriendly environmental planning and design, severe fragmentation of spatial layout, environmental pollution, garbage disposal, and other issues that cannot keep up with the pace of development. Among these，the site selection of the waste incineration power plant is a significant problem. Hydrogeological conditions, service areas and operation capacity, environmental protection evaluation, process equipment, and other factors are critical criteria for consideration in the current site selection of waste incineration power plants. However, the possible actual impact on surrounding residential areas, especially the risk of psychological impact on residents, is not considered enough. In the process of site selection, government departments often make “black box” decisions on behalf of the people, and the factory is silent about the negative externalities of waste incineration. In minimal risk communication, once the site selection event starts to enter the public’s view, under the combined effect of risk factors, interest factors, psychological factors, trust factors, etc., it is easy to cause people to overreact to “acceptable risk” and “pollution threshold,” In turn, it is possible to take the surrounding residents as the leading force, gather against building “In my backyard,” and initiate various forms and degrees of neighbor avoidance resistance. Thus, a series of standardized processes and procedures established for the construction and run of WIPs cannot satisfy the residents. For example, residents have protested against the construction of waste incineration plants in several cities in recent years, including Panyu, Guangdong in 2009, Jiufeng village, Hangzhou in 2014, Haiyan，Zhejiang in 2016, Wanning, Hainan in 2016, and Qingyuan, Guangdong in 2017. In recent years, many waste incineration projects in China have been shelved due to the opposition of nearby residents (Cheng and Liu, 2017). The successful siting of waste incineration plants heavily depends upon residents’ cooperation. Thus, a nuanced understanding of responses from local communities close to waste incineration plants is becoming an important research and policy focus (Huang et al., 2015; Xu and Lin, 2020).

The factors influencing public attitudes toward waste incineration plants are multi-dimensional, including political, socio-economic, and physical characteristics (Hunter and Leyden, 1995; Baxter, 2006; Dodds and Hopwood, 2006; Liu et al., 2019). Although the existing research has primarily focused on the predictors of public attitudes and other determinants of social responses to waste incineration plants, it does not adequately capture the heterogeneity of public perceptions of waste incineration plants. As some scholars have suggested, local “publics” and their understandings of locally unwanted facilities should not be perceived in an undistinguishing manner (RSS, 1992). The factors affecting local acceptance of such facilities may vary according to individuals’ profiles and perceptional diversifications. Recognizing this, some studies have used the Q methodology to explore the subjective perceptions of individual residents in the vicinity of unwanted local facilities, such as wind farms (Frate et al., 2019) and nuclear power plants (Venables et al., 2009). Q methodology requires participants to rank a set of statements on a given issue by considering all the comments simultaneously, thus revealing their subjectivities, such as the likes and dislikes of the participant group on specific aspects of the topic (Lee, 2017). This study employs the Q methodology to analyze the diversity of perceptions of residents on the siting and establishment of waste incineration plants in China, using Xuzhou No.2 waste incineration plant as a case study. The findings demonstrate that taking the deep-seated values and perspectives among hosting residents’ into consideration is vital for the siting of waste incineration plant and could inform the tailored policy to enhance favorable attitudes and public acceptability of waste incineration plants in China and elsewhere. In the next section, we review the factors influencing social responses to the siting of waste incineration plants. Then, we depict the methods for data collection and analyzing strategy. Finally, results are demonstrated and discussed.

## Literature review

Some scholars have addressed public attitudes towards waste incineration plants, and confirm that perceived risks and economics, social justice, social capital, political efficacy, place attachment, and knowledge possessions are pivotal factors in shaping public responses. This section provides an overview of the existing literature on the factors that shape community attitudes and responses to the siting of waste incineration plants.

### Perception of risk and economics

People’s opposition to incinerators is deeply rooted in the concern that the construction and operation of waste incineration plants will produce adverse effects on the local environment, public health, and the local economy (Petts Management JJW and Research, 1992; Hunter and Leyden, 1995; Porteous, 2001; Achillas et al., 2011). Generally, waste incineration could generate harmful substances such as dioxin, wastewater, landfill leachate, ash, and dust (Ren et al., 2016; Hou et al., 2019). Residents living near waste incineration plants could be exposed to awful smells and noises (Dodds and Hopwood, 2006; Ren et al., 2016). Moreover, research has shown a positive association between the location of incineration facilities and the incidence of abnormal childbearing, congenital abnormalities, and infant mortality (Subiza-Pérez et al., 2020). Cancer and other chronic diseases have also been associated with incineration facilities’ location (Comba et al., 2003). Waste incineration plants have also been stigmatized because the adverse impacts on the environment and public health could, in turn, lead to economic consequences, such as a decline in real estate values and a general decline in the ability of the host communities to attract financial investment (Lober, 1993). People living near a waste incineration plant may be worried that their property values will decline, although such concerns are often masked by environmental and health problems (Morell and David, 1984). These potential negative impacts of waste incineration plants have been found to influence the public’s perception of risk and economics and hence, their attitudes towards waste incineration plants (Wester-Herber, 2004). A large number of studies have proved that it is the enhancement of perceived risk and the attenuation of perceived economics that lead to the strong opposition of local communities to the siting and operation of waste incineration plants (Achillas et al., 2011; Baxter et al., 2016).

### Social justice

Apart from perceived risk and perceived economics, existing studies have examined public attitudes to waste incineration plants from other perspectives. Research shows that perceptions of residents on social justice about waste incineration plants, such as distributive justice, interactive justice, and procedural justice, influence their attitudes and responses (Morell and David, 1984; Lober and Green, 1994; Venables et al., 2009; Frate et al., 2019). Distributive justice refers to the degree to which the costs and benefits of societal efforts are fairly allocated within a given society (Visschers and Siegrist, 2012). There is much empirical evidence of a strong relationship between distributive justice and public attitudes (Morell and David, 1984; Hunold and Young, 1998; Ferreira and Gallagher, 2010). Introducing waste incineration plants in a given locality may expose residents to environmental, health, and economic risks with few benefits. The uneven distribution of risks and economics contributes to residents’ opposition to such facilities. Empirical studies have found that fair resettlement and transparent compensation can effectively increase public acceptance of waste incineration plants by improving the public’s perception of distribution justice (Huang et al., 2015; Liu et al., 2019).

Interactive justice is another factor influencing public attitudes towards waste incineration plants. Interactive justice refers to the fairness and acceptability of the principles that shape interaction processes among diverse groups (Cohen-Charash and Spector, 2001; McComas et al., 2008). Local government agencies that demonstrate respect towards the public and provide timely and relevant information on waste incineration plants can maintain a relationship of trust with the public, which in turn could nurture favorable public attitudes towards waste incineration plants (Morell and David, 1984; Liu et al., 2019).

Procedural justice refers to using fair and nondiscriminatory procedures in decision-making, with particular emphasis on meaningful participation in decision-makings (Cohen-Charash and Spector, 2001; McComas et al., 2008; Rasch and Köhne, 2017). Research has shown that procedural justice strongly affects public attitudes towards waste incineration plants and other public facilities (Cohen-Charash and Spector, 2001; Besley, 2010). Some authors believe that public participation, coupled with rationality and legality in the procedures for site selection and construction of waste incineration plants, could reduce the public’s risk perception and engender a more favorable attitude towards such projects (Kasperson, 2012; Antadze, 2013).

### Political efficacy

Political efficacy refers to the perception of the influence of an individual’s political actions on the political process. It is also the perception of an individual’s ability to fulfill civic responsibilities (Campbell et al., 1954). In societies with a high level of political efficacy, people are inclined to perceive that social problems could be solved through their knowledge, ability, and efforts. They tend to be more active in public affairs (Caprara et al., 2009). Previous studies have shown that people’s political efficacy could predict the possibility of acting to protest against waste incineration power plants. A recent study found that political efficiency is an essential determinant of residents’ participation in Not in My Backyard (NIMBY) resistance activities of residents around landfills and incinerators in Hong Kong (Liu et al., 2018). Similarly, another study conducted in the United States found that political efficiency significantly positively affected people’s oppositional attitudes toward a proposed high-voltage overhead transmission line near their community (Joe et al., 2016).

### Trust and social capital

It is generally believed that the lack of trust in relevant government representatives, private enterprises, and experts undermines residents’ acceptance of waste incineration plants (Freudenburg and Rursch, 1994; Petts, 1994). Social trust refers to a person’s beliefs about other individuals or organizations in a social network regarding their competence and predictability about the behavior of interest (Kasperson, 1992). Trust can squeeze or replace external uncertainty through inner certainty, thus weakening the panic and emotional rendering caused by uncertainty in social networks. Previous work has shown that social trust heavily shapes public attitudes towards public infrastructure projects (Frewer et al., 1996; Poortinga and Pidgeon, 2005; Jenkins-Smith et al., 2011; Khammaneechan et al., 2011). It is acknowledged that when there is a lack of sufficient knowledge and information to evaluate waste incineration plants, the public generally tends to make decisions concerning integrity and competence-based trust (Johnson and Scicchitano, 2012; Liu et al., 2018). In this process, trust can affect the public’s attitude towards waste incineration plants by influencing the public’s subjective perception of risks and economics (Hamer, 2003; Elliott and McClure, 2009; Guo and Ren, 2017). Therefore, restoring and promoting trust is considered an indispensable component of promoting favorable public responses to waste incineration plants and other local infrastructure development projects (Yang et al., 2016; Liu and Wang, 2019).

### Place attachment

Place attachment is another construct that has been found to shape public attitudes toward the siting of waste incineration plants (Hou et al., 2019; Subiza-Pérez et al., 2020). Place attachment is conceptualized as emotional attachment to certain areas based on personal life experience (Zhu and Liu, 2011). Studies have found that place attachment significantly affects pro-environmental behavior and behavioral intentions (Scannell and Gifford, 2010). Individuals with a stronger attachment to their local environments tend to be more willing to participate in local affairs (Wu et al., 2019; Wang et al., 2020). Place attachment has also shaped public attitudes towards waste incineration plants in China (Hou et al., 2019).

### Level of knowledge

Locally unwanted facilities like waste incineration plants possess technological attributes. In this regard, how residents perceive and react to waste incineration plants is determined to a certain extent by their scientific and technical knowledge. Existing studies document that the outcome of perceived knowledge on public attitudes is inconsistent. Some studies have revealed that residents’ knowledge of waste incineration plants can effectively attenuate their risk perception, which has obvious advantages in reducing the public’s psychological barriers to supporting the siting of waste incineration plants (Ren et al., 2016). Other studies show that the more knowledge a person holds, the more negative the attitude toward these facilities (Wright, 1993). The mixed results between the level of knowledge and public attitudes toward waste incineration plants are consistent with findings from studies on related infrastructures, such as hydrogen gas stations and nuclear waste treatment facilities (Kraft and Clary, 1991; O’Garra et al., 2008).

## Methods, data, and analysis

### Case-study context

This paper uses Xuzhou No.2 waste incineration plant as a case study to investigate residents’ subjectivity patterns. As a central regional city in northern China, the development of Xuzhou represents the track of cities in northern China to some extent. These cities need to establish waste incineration power plants, but the economic orientation of urban development planning leads to relatively extensive decision-making on the location of adjacent facilities. In addition, through the interview, it is found that the surrounding residents have significant levels and representativeness in terms of gender, age, income and educational background, which is convenient to explore the heterogeneity of residents’ acceptance of waste incineration power plants.

The No.2 waste incineration plant is located at Jiahe, a little village in eastern Xuzhou, Jiangsu Province. Xuzhou No.2 waste incineration plant seats on the outskirts of urban areas, is close to the south side of national highway 310 and is 25 km away from downtown. The plant covers an area of about 14,667^m2^ (220 mu). The total project investment is 1.267 billion yuan, of which environmental protection investment is about 198.920 million yuan, accounting for 15.7% of the total investment. Construction of the plant officially started in June 2019. Generator sets 1 and 2 of the first stage of the Construction have been formally completed and put into operation in July and October 2020, respectively. A total of 5 sets of mechanical grate furnaces and three sets of 25 MW steam turbine generator units were installed in the project’s two phases. After the project is implemented, it can handle 1.5 million tons of domestic waste annually and generate 594 million kWh of electricity annually. The operation of the NO.2 waste incineration plant suggests Xuzhou City will terminate landfills to dispose of municipal solid wastes.

### Q-methodology

Q methodology is employed to explore the diverse perceptions of residents on the establishment of waste incineration plants considering its advantage in capturing subjectivities (Rajé, 2007). The purpose of the Q methodology is to conduct a rigorous quantitative analysis of the subjective perceptions of a group of participants on an exciting issue. Quantitative data for the Q methodology is generated by asking respondents to rank a set of statements on a given subject matter based on their meanings, interpretations, likes, dislikes, and other subjectivities. The collected data are then calculated using appropriate statistical procedures, such as factor analysis, to reveal the viewpoints expressed by various groups of respondents (Watts and Stenner, 2005). Thus, the Q methodology combines the advantages of both qualitative and quantitative approaches (Dennis and Goldberg, 1996). Q methodology is also based on the assumption that a limited number of opinions exist on a given topic in society and that the views of a small number of subjects with different backgrounds are sufficiently representative of the ideas in the population of interest (Brown, 1980). The existing literature mainly focuses on the factors influencing residents’ acceptance, aiming to determine the influence of different factors on residents’ acceptance. However, this does not fully reflect the overall subjective view of residents on the waste incineration power plant, especially their overall view of the location of adjacent facilities and the heterogeneity in it. This study aims to sort out the perspectives on the local acceptance of waste incineration plants rather than focusing on the net impact of a single factor on local acceptance. The Q methodology could help identify the areas of consensus and disagreement in residents’ perceptions about waste incineration plants.

### Q-set generation

A critical step in applying the Q methodology is developing a statement that provides comprehensive coverage of the exciting issue, i.e., the Q-set. It is through the respondents’ ranking of these statements that subjectivity is assessed (Watts and Stenner, 2005). Following previous suggestions (Watts and Stenner, 2005), statements for the Q-set in this study were generated through interviews focusing on public responses and acceptability of waste incineration plants 22 residents and a review of the relevant literature. Following Watts and Stenner (2005), many statements were initially generated from the interview data. A final Q-set, composed of 39 statements, was generated after refining and reducing the initial list of statements. These statements cover a variety of categories and sub-categories representing perceptions on waste incineration plants: risk perception, interest, justice (procedural justice, interactive justice, distributive justice); political efficiency; social trust (local government trust, waste incineration plant trust); place attachment; and knowledge level. The Q-set employed in this study represents the theoretical and practical foundation (Zhang, 2021). The specific items of the Q statements are shown in Table 1.

**Table 1:** Q statements.

Classification	Contents
Risk perception	1. The waste incineration plant will cause air and water pollution. 2. The waste incineration plant will affect the health of residents. 3. The waste incineration plant will cause accidents such as explosions and fire. 4. The waste incineration plant will put psychological pressure on residents
Economic impacts	5. The waste incineration plant will affect the development of local agriculture, tourism, and other industries. 6. The waste incineration plant can lead to a decline in local property values. 7. The waste incineration plant can provide jobs for residents. 8. The waste incineration plant can provide better local waste treatment
Justice perception	9. We participated in the site selection and construction of the waste incineration plant. 10. The site selection and construction of the waste incineration plants are reasonable and legal. 11. Environmental impact assessment of the local waste incineration power generation project is carried out according to legal procedures
	12. The government officials I deal with are always friendly and polite. 13. Government officials value my rights. 14. The local government inform me of the site selection and construction of the waste incineration plant and give a full explanation promptly. 15. The local government attaches great importance to my opinions and questions about the waste incineration plant and gives a patient explanation
	16. Residents near the local waste incineration plant have received some compensation. 17. The local waste incineration plant provides convenience for the waste treatment of the whole society, but its negative impact is borne by the surrounding residents
Political efficacy	18. I have a strong understanding of public issues. 19. I feel able to participate in public affairs. 20. The government can take my opinion seriously. 21. Government officials care more about people’s ideas
Social trust	22. Local governments are concerned about the interests of the people. 23. The local government does not conceal information or deceive the people. 24. Local governments can effectively supervise public facilities such as waste incineration plants. 25. Local governments can formulate science-based and reasonable policies
	26. The managers of the waste incineration plant care about the interests of the people. 27. The managers of the waste incineration plant do not conceal and cheat the familiar people. 28. The managers of the waste incineration plant have rich experience in the development and operation of waste incineration power generation projects. 29. The managers of the waste incineration plant have professional skills in developing and operating waste incineration projects
Place attachment	30. I do not want to relocate from my current place of residence. 31. This place can meet all my needs. 32. My friends and relatives are in close geographical proximity, and I can keep in touch with them. 33. I have a sense of belonging to this place. 34. This place means a lot to me. 35. For me, there’s no other place like this place
Knowledge	36. When there is a waste incinerator, the landfill is not necessary. 37. When the temperature in the incinerator is about 900°C, the combustible and harmful components in the waste will be decomposed. 38. Waste sorting is the premise of waste incineration. 39. Wet waste such as leftovers cannot be directly burned in the waste incinerator

### Participants

**Figure 2:**
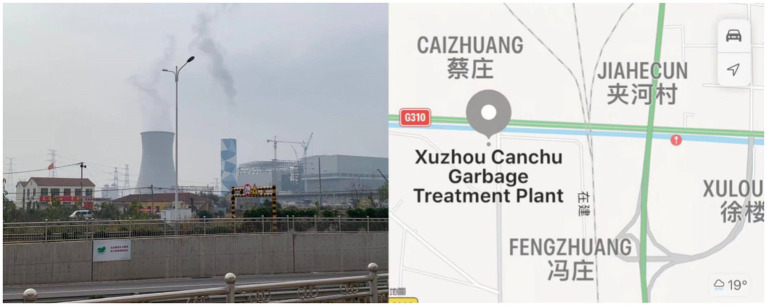
Xuzhou No. 2 waste incineration plant and its location (Photo taken by YH and ZZ).

**Figure 3:**
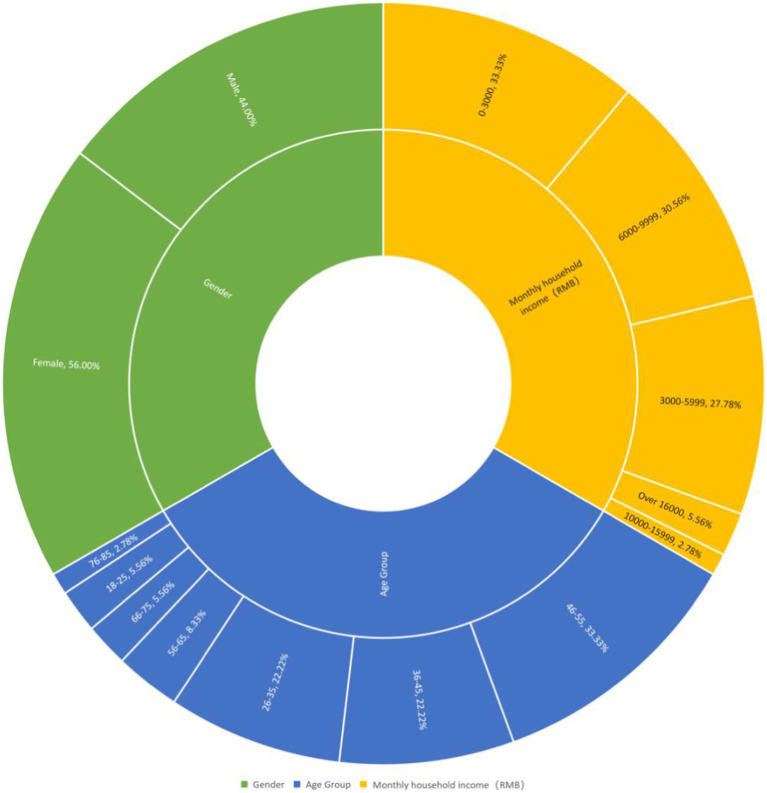
Participants’ information (percentage %).

The data for the study were collected in November 2020. The respondents are permanent residents from Jiahe Village, within 1 km from the waste incineration plant. According to the regulations in China, waste incineration plants shall not be located in built-up areas of large and medium-sized cities (Ministry of Ecology and Environment of the People’s Republic of China, 2008). Most of China’s waste incineration plants are located on the rural–urban fringe. The Xuzhou No.2 waste incineration plant is in a rural area close to Xuzhou City. To ensure representativeness and diversity of residents’ rural and urban characteristics in the study area, the survey adopts snowball sampling and purposeful sampling to guarantee respondents’ proper distribution in gender, age, education background, occupation, and income as far as possible. Finally, 36 persons took part in the survey (Figures 2, 3).

### Q-sorting

Q-sorting is the process by which data is generated for subsequent analysis in Q methodology research (Brown, 1980). In this study, we adopt the 11-level forced distribution model. After carefully comparing 39 statements in the Q-set, the respondents are asked to fill in the standard distribution table with the corresponding number of each sentence based on their interpretation of each statement and their level of agreement with it. Through this procedure, participants rank the statements in the Q-set using the distribution table provided (Table 2). An 11-point scale, ranging from +5 to−5, with a ranking of +5 representing a solid level of respondents’ agreement with the statement. In contrast, a hierarchy of-5 represents a strong disagreement with the statement. To make it easier for the respondents to rank each statement, they are first asked to divide the 39 statements into three categories: approval, neutrality, and disapproval. Then, they were introduced to rank statements in each of the three categories using the distribution table until they were satisfied with the placement of each sentence. Finally, the follow-up interviews are conducted randomly with several participants to further dig into the explanations behind their statement placement. Each interview lasts around 40 ~ 50 min.

**Table 2:** Normal distribution.

Least like my point of view								Most like my point of view		
-5	-4	-3	-2	-1	0	1	2	3	4	5
										
										
									
						
				
		


### Data analysis

Special statistical software for Q methodology, PQ Method 2.35, is used to analyze the Q classification data. Principal component analysis with varimax rotation is employed to identify the factors embedded in the data. Factors with eigenvalues greater than 1, with at least two sorts of loading, significantly satisfy the demands of factor selection (Brown, 1980). With the recommendation from Watts and Stenner, the loading value of significant sorts is computerized as 2.58*(1/$\sqrt{\mathrm{No}}.\mathrm{of items in}\;Q\;\mathrm{set}$). Therefore, the effective threshold for the present study is ±0.48.

Initially, six factors are extracted with two structures with only one significant sort, which theoretically does not make sense. Four sorts load significantly across more than one factor, which was excluded for further analysis considering factor purity (Watts and Stenner, 2005). Finally, four factors emerged from the study, with all the loadings greater than 0.48 (Table 3). These four factors account for 27, 8, 23, and 9% of the variance, respectively, cumulatively explaining 69% of the total variance.

**Table 3:** Extract factors from the rotated factor matrix.

Group participant	Factor 1	Factor 2	Factor 3	Factor 4
1	**0.72**	0.021	0.33	0.29
2	**0.64**	0.07	0.29	0.33
3	**0.79**	0.19	0.31	0.14
4	**0.79**	0.16	0.17	0.03
7	**0.56**	0.17	0.47	0.13
14	**0.63**	0.14	0.42	0.15
17	**0.85**	0.10	0.25	0.15
21	**0.76**	0.27	0.38	0.10
27	**0.78**	0.18	0.29	0.20
29	**0.70**	0.10	0.36	0.26
30	**0.63**	0.18	0.42	0.25
31	**0.74**	0.03	0.25	0.34
34	**0.73**	0.21	0.34	0.23
11	0.21	**0.52**	0.35	0.42
28	0.13	**0.90**	0.11	0.02
5	0.47	0.31	**0.57**	0.13
6	0.30	0.07	**0.56**	0.45
12	0.35	0.42	**0.50**	0.30
13	0.40	0.21	**0.64**	0.17
15	0.35	0.36	**0.53**	0.28
16	0.22	0.09	**0.87**	0.10
18	0.42	0.32	**0.61**	0.20
20	0.48	0.10	**0.56**	0.20
24	0.28	−0.10	**0.80**	0.20
25	0.37	0.38	**0.64**	0.22
26	0.42	0.21	**0.56**	0.33
35	0.30	0.36	**0.69**	0.34
36	0.40	0.14	**0.61**	0.09
23	0.36	−0.01	0.20	**0.76**
33	0.28	0.45	0.23	**0.64**

Bold values here refers to the factor loadings is meaningful.

## Results and discussion

Following the practices by Watts and Stenner (2005), factor interpretation in the present study is supported by two pieces of evidence. The interpretation first looks at the ‘characterizing’ statements, which place around both extremes of a social perspective (factor). The interpretation then turns to the distinguishing statements, which display the uniqueness of a given factor and differentiate the given factor from others. The interview materials over the Q sorts collection are also utilized to enrich the interpretation of the social perspective through in-depth analysis. The four perspectives are labeled according to extreme item statements loaded and interview materials. Table 4 presents the statements with extreme Q-sort values and their corresponding z-scores of each perspective. Statements with * are the distinguishing statements of each perspective.

**Table 4:** Overview of the four perspectives and their extreme item statements with normalized Q-sort values and z-scores.

Statements	Q-sort value	z-scores
Factor 1: I do not trust them and feel besieged by risks		
2. The waste incineration plant will affect the health of residents*	5	2.082
1. The waste incineration plant will cause air and water pollution*	5	1.952
3. The waste incineration plant will cause accidents such as explosions and fire	4	1.558
17. The local waste incineration plant provides convenience for the waste treatment of the whole society, but its negative impact is borne by the surrounding residents	4	1.521
26. The managers of the waste incineration plant care about the interests of the people	−4	−1.362
27. The managers of the waste incineration plant does not conceal and cheat the familiar people	−4	−1.392
23. The local government does not conceal information or deceive the people	−5	−1.479
22. Local governments are concerned about the interests of the people	−5	−1.492
Factor 2: I trust local governments but I am unfairly treated		
21. Government officials care more about people’s ideas	5	1.751
22. Local governments are concerned about the interests of the people*	5	1.751
13. Government officials value my rights*	4	1.453
25. Local governments can formulate science-based and reasonable policies*	4	1.295
14. The local government inform me of the site selection and construction of the waste incineration plant and give a full explanation promptly	−4	−1.559
26. The managers of the waste incineration plant care about the interests of the people	−4	−1.612
16. Residents near the local waste incineration plant have received some compensation.	−5	−2.015
9. We participated in the site selection and construction of the waste incineration plant	−5	−2.015
Factor 3: I attach this place a lot but I am unfairly treated		
30. I do not want to relocate from my current place of residence	5	1.720
34. This place means a lot to me	5	1.737
33. I have a sense of belonging to this place*	4	1.636
35. For me, there’s no other place like this place	4	1.347
21. Government officials care more about people’s ideas	−4	−1.563
22. Local governments are concerned about the interests of the people	−4	−1.469
9. We participated in the site selection and construction of the waste incineration plant	−5	−1.712
16. Residents near the local waste incineration plant have received some compensation	−5	−1.585
Factor 4: I possess knowledge of waste incineration and feel besieged by risks		
1. The waste incineration plant will cause air and water pollution	5	1.658
2. The waste incineration plant will affect the health of residents	5	1.820
4. The waste incineration plant will put psychological pressure on residents	4	1.553
39. Wet waste such as leftovers cannot be directly burned in the waste incinerator	4	1.553
38. Waste sorting is the premise of waste incineration	3	1.477
37. When the temperature in the incinerator is about 900°C, the combustible and harmful components in the waste will be decomposed	−5	−1.714
36. When there is a waste incinerator, the landfill is not necessary	−5	−1.981

* indicates distinguishing statement.

### Factor 1: I do not trust them and feel besieged by risks

Factor 1 possesses an eigenvalue of 21.31 and is the dominant perspective with 27% of the total variance. Thirteen sorts are significantly loaded on this factor, which comprises three males and 10 females. Participants classified into this perspective observes that waste incineration plants could incurs adverse hazards and significant risks. Participants of this viewpoint argued that waste incineration power plants would cause air and water pollution (s.1*:+5). Respondent 1 is a pregnant private owner. She said: “*Before the construction of the waste incineration plant*, *all we used here was groundwater*, *and the water was so sweet*. *Now the people in the factory (waste incineration plant) leach out the harmful waste*. *The liquid was secretly poured into the ditch next to our village*, *and the groundwater that came up was covered with a layer of oil*, *and it was impossible to drink*.” Being pregnant, she was forced to buy bottled mineral water at a high price for daily drinking since she worried that polluted groundwater would affect the development of the fetus.

Participants believe that severe pollution of air and water led by waste incineration is borne by the surrounding residents (s.17:+4) and that the health of nearby residents will inevitably be undermined by environmental pollution (s.2:+5). Other residents represented by respondent 2 said, “*In summer*, *the smell in the air is too stinky*, *pungent*, *and even worse than dung*. *The pungent smell cannot be blocked by closing the doors and windows*, *which would enhance the possibility of respiratory and skin disease*.” This concern is also ignited by the situation in neighboring villages. The neighboring village is a chemical factory base, where the air and water are polluted. “*The chemical plant built in the neighboring village has seriously polluted the air and water there*. *As a result*, *many residents there have got cance*r” (Participant 7).

Meanwhile, participants from this perspective perceive waste incineration plants can also be exemplified by safety accidents such as explosions and fires (s.3:+4).There is also skepticism about the values and deeds of waste incineration plants and local government agencies. Participants structured within this perspective have a nasty evaluation of the credibility of local government and think that the local government is not credible, including not only not caring about the interests of the people (s.22:-5) but also concealing and deceiving the people (s.23: −5). These views mainly depend on the government’s closed policy-making model, the practice of prevarication between the superior and the lower levels and various departments, and the ignoring and perfunctory attitude towards the problems raised by neighboring residents. As respondent 4 said: “*We went to the town government to ask for an explanation*. *The town government responded that they understood our situation well but were also powerless*. *The town government suggested we wait for the research conclusions of the district government or complain to the environmental protection department*. *So*, *we complained to the Environmental Protection Agency*. *The EPA staff pretended to collect water samples for testing*, *but it had been several months*, *and no test results were given to us*. *Government officials are always evasive and shielding*.” Compared with the extreme distrust of the local government, their negative trust perception towards the waste incineration plant is not much higher. They believe that the waste incineration plant not only does not care about the interests of the people (s.26:-3) but also has the possibility of concealing and deceiving the people (s.27:−4). Participants’ non-trust in the plants hinges heavily on their personal experience. Respondent 3 mentioned an incident that made them feel aggrieved and concerned: “*we went to the gate of the waste incineration plant to ask for an explanation*, *but the plant staff called the police*, *and then all of us were arrested*.” Some studies support the view that social trust will affect people’s attitudes towards risk (Flynn et al., 1992). People will rely on trust to judge to reduce the complexity of risk management decisions (Slovic et al., 1991). According to this logic, the loss of social confidence will intensify people’s risk perception, which will cause this group to oppose waste incineration power generation projects strongly.

### Factor 2: I trust local governments but I am unfairly treated

Factor 2 has an eigenvalue of 2.09 and is found to explain 8% of the study variance. Two sorts consisting of two females load significantly on this factor. The two participants display high trust in the local government. They state that the local government cares about the people’s interests (s.22:+5) and that local government can formulate scientific and reasonable policies (s.25:+4). As respondent 28 pointed out: “*We watch the news every day*. *Too many reports show that the government is a good government that serves the people*. *Moreover*, *the government has done many practical things that care about the people*. *For example*, *in our village*, *the government has been sending people to clean the home for free for the elderly over 80*. *Therefore*, *I believe that the government has carefully considered the construction of the waste incineration plant here*.” It can be seen from the statements that media communication plays a critical role in cultivating and enhancing people’s trust in government agencies (Che, 2019). In addition, both of the participants are members of the Communist Party of China (CPC). Therefore, there is reason to believe that the membership of the CPC, effective party member education, and the expectations in the governing ability of the CPC maintain their trust in local governments. Considering the affiliation and connections with CPC, both show solid political efficacy, believing that government officials care more about the people’s ideas (s.21:+5) and value their rights (s.13:+4).

However, to some extent, their solid political efficacy becomes weird and illusionary regarding their unjust experiences and treatment of waste incineration plants. The statements that rank most negatively for this perspective are those centered around justice and equity, participation in the site selection and construction of the waste incineration plant (s.9:-5), and complete explanation and site selection of the waste incineration plant (s.14:-4). More importantly, the absence of compensation for residents of the host community (s.16:-5) intensifies the interests deprivation concerning waste incineration plant does not care about the interests of the people around (s.26:-4). As participant 11 said, “*A few years ago*, *coal mines came to us to mine coal and compensated more than 30 million in our village*. *This time the waste incineration plant should also compensate us like the coal mine*, *or it is a terrible injustice for us*.” This part of the group will always compare the lack of compensation from the waste incineration plant with the compensation package from coalmines and argue that the waste incineration plant ignores their interests.

### Factor 3: I attach this place a lot but I am unfairly treated

Factor 3 has an eigenvalue of 1.20, accounting for 23% of the variance. This factor is significantly defined by 13 sorts, comprising five females and eight males. Similar to factor 2, the participants structured within factor 3 indicate that local governments and firms running waste incineration plants also unfairly accommodate them. As respondent 13 pointed out: “*No one ever told us to build a waste incineration plant*. *The government did not tell us about it when it expropriated our land*. *We did not know until a wall was built not far away*,” he is not recruited to participate in the public meeting about site selection and construction of waste incineration plant (s.9:-5). Furthermore, the participants are highly dissatisfied that there is no compensation plan for nearby residents (s.16:-5). Respondent 20 said: “*The government expropriates our land by leasing to build a waste incineration plant but has not paid us the rent up to now*.” The absence of compensation forges their inclination that local government agencies and officials do not value their interests (s.22:-4).

The most notable statements of these participants are that they have a strong sense of local attachment. They put almost all expressions of the local affirmative attachment in the table with the highest score. They made it clear that they did not want to leave their current living environment (s.30:+5), this place is of great significance to them (s.34:+5), and they have a sense of belonging to this place (s.33:+4), there is no other place like this (s.35:+4), there are relatives and friends here to keep in touch (s.35:+4). Participant 16 said: “*I have such a big house and yard now*. *I am used to living and feel it is not as good as here*. *My brother bought a house in the town*. *The house is small*, *and there is no place for cars to park*. *Some people in the village have moved away because of the pollution caused by the waste incineration plant*, *but I do not want to go*. *If this place is not demolished*, *I will stay here*.” Several participants suggest they feel comfortable living here. Even if the government arranges relocation plans for them to town as a whole without any personal costs, they are not willing to go (Participants 13,18,24). Without pre-public inquiry, the rash construction of a waste incineration plant makes residents feel the construction and operation of the waste incineration plant have destroyed their emotional dependence on the physical space of their living environment (Devine-Wright, 2009). As respondent 26 commented: “*This is my home*, *where I was born and raised*. *They set up a waste incineration plant here is equivalent to putting cancer in my home*. *I have the responsibility and obligation to expel this bad thing*. *Even if the expelling is unsuccessful*, *I will fight to the end and show my attitude*. *I participated in the previous protests every time*, *and I will be more active in the future*.”

### Factor 4: I possess knowledge of waste incineration and feel besieged by risks

Factor 4 has an eigenvalue of 1.07 and is found to explain 9% of the total variance. Two significant sorts are classified into this factor, which are both males. This perspective is characterized by waste incineration knowledge and perception of risks. On the one hand, Factor 4 reflects that the participants perceive the vital risks as factor 1. They are very affirmative about the air and water pollution caused by waste incineration plants (s.1:+5) and the health of residents (S.2:+5). Apart from these physical impacts, they also insist that the waste incineration plant will cause psychological stress to residents (s.4:+4), which could be evidenced by the words “*I felt uncomfortable when I saw the smokestack in the yard*” (Participant 23).

On the other hand, their understanding of waste incineration reveals their concern for the extensive management of current municipal solid management in China. They feel that waste incineration is subject to some social conditions, like garbage sorting (s.38:+3), and wet waste, such as leftovers, cannot be directly burned in a waste incinerator (s.39:+4). When there is a waste incinerator, the landfill is not necessary (S.11:-5). To some extent, the subjectivity regarding these statements reflect their objective knowledge level about waste incineration. Some people contend garbage could be economically recycled and then incinerated to avoid excessive harm. As Participant 23 said: “*Leftovers are used to feed pigs*, *and they are wasted when they are burned in a waste incinerator*.*”* Furthermore, their somehow non-objective internalized knowledge also can be revealed with even if the temperature in the incinerator reaches 900°C, the combustible and harmful components in the waste will not be decomposed (s.37: −5). As Participant 33 said: “*burning waste certainly cannot decompose harmful components*. *If it can decompose*, *how can there be black smoke and odor?*” After that, he affirmed without hesitation: “*the propaganda that waste incineration can decompose harmful components must be a lie made up by the waste incineration plant for its construction and smooth operation! They can do everything for their economics*.”

## Conclusion and policy implications

Underpinned by the post-positivist paradigm, this paper takes a case study of a waste incineration plant sitting in Xuzhou, China. It applies Q-Methodology to explore a pressing policy issue of public acceptance. Thirty-nine representative statements are constructed based on interview materials and literature review. This study identifies four configurable perspectives: I do not trust them and feel besieged by risks; I trust local governments but I am unfairly treated; I attach this place a lot but I am unfairly treated; I possess knowledge of waste in-cineration and feel besieged by risks. Our findings reveal that each individual’s position is profoundly shaped by personal and collective values embedded within social-economical contexts, highlighting the inadequacy of the NIMBYism framework (Wolsink, 2006). Our research provides an example of how Q-Methodology can be applied to the siting of a waste incineration plant. It positively responds to the call that more theoretical-foundation research and the usage of greater methodological diversity can well explain how public acceptance is constructed concerning locally unwanted facilities (Devine-Wright, 2005).

The analysis of the public acceptance of waste incineration in Xuzhou provides insight into the siting policy impasse regarding the waste incineration plant. Successfully siting up waste incineration plants depends on public acceptance is becoming the consensus of the whole society. To realize this consensus, inclusive governance and an effective public consultation mechanism are musts. Within a traditionally strong government but a weak society, local government agencies show their powerful disdain for including the public in the decision-making, which adds fuel to the fire for the sitting waste incineration plant. Thus, local government agencies should adjust their values and mindsets and keep in mind the unsustainability of the siting policy without the inclusion of residents. The smooth operation of inclusive governance conditions on the nature of civil society, which may occur in cities with developed economies (Zhang, 2014; Li et al., 2016; Zeng et al., 2019). Therefore, the inclusive governance incurred by the public for the siting of waste incineration plants is somehow challenging in North China. Effective public consultation mechanisms should be interactive, which can change parties’ attitudes, reduce the arrogance of the opposition and supporter and cultivate trust between them. The case of Xuzhou indicates that the siting decision-making system is closed, and multiple forms of participation should be adopted. In such cases, special attention should be paid to distinct views framed in varied ways by different residents to accommodate their feedback and enhance their participation efficacy. The compensation package is an indispensable strategy to promote acceptance by hosting a community of waste incineration plants. The implementation of compensation policy request local governments to consult adequately with hosting residents, or the ‘bribery effect’ stands out.

As far as we know, this study is the first to use the Q-method to examine the acceptability of residents to shelter facilities in China’s green development context. However, this study has some limitations. The limitation of the Q-method is that it only applies to one case, so its conclusion is challenging to be extended to a larger population, which is why we chose Xuzhou, a city with green and livable characteristics, as the case site, to be more representative. However, more cases need to be studied in the future to determine whether the findings of this study have been confirmed in the future. It is also possible to conduct more in-depth longitudinal research to depict the development process of residents’ acceptability in the location decision of adjacent facilities and how communication, participation, and acceptability develop together over time to better understand the nature of the acceptability of adjacent facilities.

## Data availability statement

The raw data supporting the conclusions of this article will be made available by the authors, without undue reservation.

## Ethics statement

The studies involving human participants were reviewed and approved by School of Management, Xuzhou Medical University. The patients/participants provided their written informed consent to participate in this study.

## Author contributions

YH and YZ: conceptualization, formal analysis, writing-original draft, visualization, and project administration. ZZ and YH: methodology and investigation. YH and YZ: software, data curation, and funding acquisition. ZZ, YH, and YZ: validation and resources. YZ: writing—review and editing and supervision. YZ and ZW: revision. All authors have read and agreed to the published version of the manuscript.

## Funding

This work was supported by Hebei Province Social Science Development Research Project (No. 20220202088), Humanities and Social Science Research Project of Hebei Education Department (No. BJS2023204).

## Conflict of interest

The authors declare that the research was conducted in the absence of any commercial or financial relationships that could be construed as a potential conflict of interest.

## Publisher’s note

All claims expressed in this article are solely those of the authors and do not necessarily represent those of their affiliated organizations, or those of the publisher, the editors and the reviewers. Any product that may be evaluated in this article, or claim that may be made by its manufacturer, is not guaranteed or endorsed by the publisher.
